# Asymptomatic Carotid Disease and Cognitive Impairment: What Is the Evidence?

**DOI:** 10.3389/fneur.2021.741500

**Published:** 2021-11-18

**Authors:** Hediyeh Baradaran, Amir Hossein Sarrami, Ajay Gupta

**Affiliations:** ^1^Department of Radiology and Imaging Sciences, University of Utah, Salt Lake City, UT, United States; ^2^Department of Radiology, Weill Cornell Medicine, New York, NY, United States; ^3^Feil Family Brain and Mind Research Institute, Weill Cornell Medicine, New York, NY, United States

**Keywords:** carotid atherosclerosis, cognitive impairment (CI), dementia, carotid stenosis, carotid plaque (CP)

## Abstract

The development of cognitive dysfunction and dementia is a complex, multifactorial process. One of the contributors to various types of cognitive dysfunction is carotid atherosclerosis which can frequently be seen in asymptomatic individuals. There are a number of different manifestations of asymptomatic carotid atherosclerosis including arterial stiffness, carotid intima-media thickening, flow-limiting stenosis, and complex, atherosclerotic plaque. Each of these forms of atherosclerosis may contribute to cerebral parenchymal damage, contributing to cognitive dysfunction. In this review article, we will discuss each of these forms of carotid atherosclerosis, present the potential mechanistic underpinnings behind an association, and then review the scientific evidence supporting potential associations to cognitive dysfunction and dementia.

## Introduction

With an ever-increasing aging world population, there is increasing demand for identifying effective preventative and treatment strategies for the development of dementia and cognitive dysfunction (1). Early identification and effective treatment of dementia and cognitive dysfunction has been an ongoing challenge due to the multifactorial nature of disease development. One of the factors that appears to be contributing to the development of cognitive dysfunction and dementia is carotid atherosclerotic disease, including carotid stiffness, increased carotid intima media thickness, flow-limiting carotid stenosis, and high-risk carotid plaque features (2, 3). While traditionally thought to primarily contribute to ischemic stroke, there is increasing evidence of the contribution of carotid atherosclerotic disease to the development of cognitive impairment and dementia. Though the exact mechanisms by which each of these manifestations of carotid atherosclerosis contribute to the development of dementia is still under investigation, they each appear to contribute in unique but perhaps overlapping ways. In this article, we will review the scientific evidence supporting the links between each of these disease processes and the development of cognitive dysfunction.

**Figure 1:**
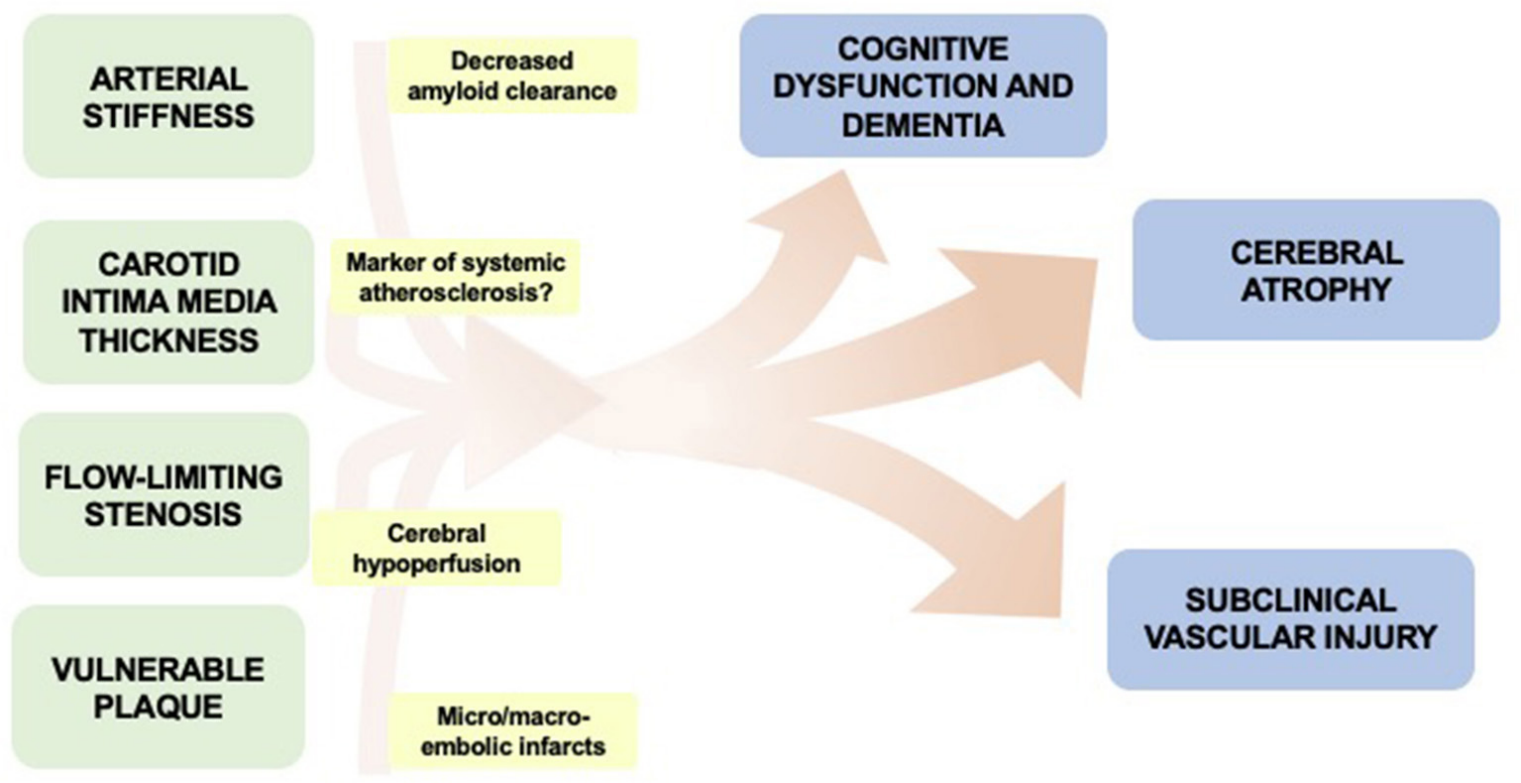
Overview of potential associations between manifestations of asymptomatic carotid disease and cognitive impairment.

Though there are many contributing factors in the development of dementia, in this review, we will focus on the role of asymptomatic carotid artery atherosclerosis in contributing to cognitive dysfunction and dementia (Figure 1). Asymptomatic carotid artery disease, which is more commonly seen in male patients, is frequently associated with vascular risk factors such has hyperlipidemia, diabetes, smoking, and hypertension. Manifestations vary, often starting as minimal wall thickening and then ultimately leading to flow-limiting stenosis and/or vulnerable plaque components which may rupture leading to cerebral ischemia. Currently, there is clinical equipoise with regard to optimal treatment for asymptomatic carotid atherosclerotic disease due to difficulties in balancing the risk and benefit calculations with treatment. Though the primary concern with carotid atherosclerosis is ischemic stroke, we will review some of the associations of asymptomatic carotid atherosclerosis to cognitive dysfunction and dementia.

First, we will discuss the often clinically silent presence of carotid stiffness and its association with cognitive impairment. We will then review a common finding indicative of subclinical atherosclerosis—carotid intima-media thickening. Then we will discuss the evidence supporting an association between flow-limiting extracranial carotid stenosis and cognitive impairment. Finally, we will review the relevant evidence behind specific carotid plaque features in the development of mild cognitive impairment and dementia.

## Search Methods for Review

We performed a robust search of the available medical literature searching for manuscripts with key terms related to carotid atherosclerosis, carotid stenosis, arterial stiffness, and carotid plaque along with any terms related to cognitive impairment, dysfunction, or dementia. The primary search was performed using PubMed and included the use of MeSH terms. In addition, we evaluated cited references in each of the manuscripts we evaluated.

## Carotid Stiffness and Cognitive Impairment

### Definition and Measurement

Stiffening of the carotid artery or other elastic arteries is the gradual loss of elastin fibers and accumulation of stiffer collagen fibers in the media over time (4, 5). This process, which can occur independent of the development of atherosclerosis, leads to loss of the ability of vasculature to appropriately accommodate to changes in blood pressure variation (6). This loss of responsive distensibility leads to higher pulsatile pressures and eventually increased flow load experienced by cerebral microvasculature and ultimately the brain parenchyma (4).

Arterial stiffness is not routinely measured in clinical practice but has been well-studied in several epidemiologic cohort studies. It is most commonly measured via indirect methods by measurement of pulse wave velocity (PWV) (7). Pulse wave velocity is an estimation of central arterial stiffness via measurement of pressure waves in two different vascular beds, commonly the carotid and femoral arterial beds (8). This indirect measure is a surrogate for aortic stiffness and has been widely used in multiple cohort studies. In addition to these indirect measures of central arterial stiffness, there are additional methods to directly measure vascular stiffness, specifically in the carotid artery (7, 9). These are most commonly performed via ultrasound measurement techniques.

### Potential Mechanisms

Since there are many shared risk factors for the development of arterial stiffness and other common cardiovascular diseases, it can be difficult to determine the specific effects of arterial stiffness on the downstream cerebral parenchyma. One of the major vascular risk factors contributing to increased arterial stiffness is increasing age. In addition to age, hypertension, diabetes, and smoking are additional factors that seem to accelerate the development of arterial stiffness (10). A major proposed mechanism by which arterial stiffness contributes to cognitive dysfunction is through the increased flow load experienced by the cerebral parenchyma leading to end-organ damage (11, 12). The resultant damage may manifest as cerebral small vessel disease evident on brain imaging, including white matter hyperintensities, covert brain infarctions, or cerebral microbleeds. These findings of cerebral small vessel disease are also independently associated with cognitive impairment and dementia, in addition to stroke and overall mortality (13–16).

### Stiffness and Imaging Markers Associated With Cognitive Impairment

Arterial stiffness has been associated with several imaging findings which are in turn also associated with cognitive dysfunction and dementia (Table 1). Specifically, there have been several studies showing that decreased carotid compliance (or increased carotid stiffness), is associated with decreased total brain and cortical gray matter volumes and also decreased volume in the hippocampal and parahippocampal regions (11, 17, 18). In the Age, Gene/Environment Susceptibility (AGES)-Reykjavik Study in 422 participants free from cerebrovascular disease and dementia, the authors found that carotid stiffness was associated with lower whole brain (-0.127 ± 0.037 SD/SD, *p* < 0.001), gray matter (−0.079 ± 0.038 SD/SD, *p* = 0.038), and white matter volumes (−0.128 ± 0.039 SD/SD, *p* = 0.028) along with lower memory scores (11). In the SMART-MR study of 526 participants, the authors found a cross-sectional association with increased carotid stiffness and lower total brain and cortical gray matter volume (*B* = −0.24%, 95% confidence interval [CI] −0.44 to −0.04%, and B = −0.47%, 95% CI −0.75 to −0.19%) but this association was no longer significant when evaluated prospectively after a mean of 4 years (17). When evaluating 614 participants in the Atherosclerosis Risk in the Community Study (ARIC), authors found a significant association between decreased carotid stiffness and lower parahippocampal and hippocampal volumes 20 years later [*R* = 0.218(0.144–0.291), *p* < 0.001 and *R* = 0.181 (0.105–0.255), *P* < 0.001], even after adjusting for confounders. Decreased total brain volumes and decreased volumes in the parahippocampal and hippocampal regions are independently associated with cognitive dysfunction and dementia (19).

**Table 1:** Overview of feature of asymptomatic carotid atherosclerosis and their potential association with cognitive dysfunction and dementia.

**Atherosclerosis feature**	**Imaging features**	**Potential mechanistic association with dementia**	**Supportive studies**
Arterial stiffness	Central: Generally measured via pulse wave velocity Carotid: Generally measured directly with Ultrasound (US) based measurement of the carotid artery	Lack of vascular distensibility leads to increased flow load experienced by cerebral parenchyma	*Central* arterial stiffness may relate to cognitive impairment (24–32) *Carotid* stiffness is associated with cognitive impairment (33–38)
Carotid intima-media thickening	Measured via US in either the distal common carotid or proximal internal carotid artery	Similar mechanism as arterial stiffness and marker for generalized cardiovascular risk	Cross-sectional (39–43) and prospective (44–46) association between increased CIMT and cognitive impairment
Flow-limiting stenosis	Measured on either US, CT angiography, MR angiography, or digital subtraction angiography. Most commonly graded using NASCET criteria	Hypoperfusion from flow-limitations or potentially increased small infarctions	Association of flow-limiting stenosis to cognitive impairment (47–54); Changes to cognition after revascularization (55–59)
High-risk plaque features	Various features indicate “higher risk” including the presence of intraplaque hemorrhage, lipid-rich necrotic core, plaque ulceration, or increased plaque thickness/volume	Increased small covert brain infarctions or other markers of cerebral small vessel disease	High plaque volume and/or vulnerable plaque is associated with cognitive impairment (60–66)

In addition to decreased cerebral parenchymal volumes, there is evidence of the association between carotid stiffness to imaging findings of cerebral small vessel disease, including white matter hyperintensities, covert brain infarctions, and cerebral microbleeds, including in the AGES-Reykjavik, SMART-MR, and ARIC epidemiologic cohort studies (20–22). In the SMART-MR study, there was a significant association between carotid stiffness and larger volume of white matter hyperintensities (*B* = 0.09%, 95% CI −0.01 to 0.19%) as well as cortical and subcortical brain infarcts (RR = 1.44, 95% CI 1.14–1.81). In the ARIC study, this association was found to be significant 20 years after the carotid stiffness measures (20). These imaging markers are also independently associated with mortality, stroke, cognitive dysfunction, and dementia (14, 16, 23).

### Stiffness and Cognitive Impairment

Though there is fairly robust evidence of an association between carotid stiffness to brain imaging markers that are associated with dementia, the association of stiffness to cognitive impairment and dementia are more clinically relevant. There are several strong studies demonstrating that central arterial stiffness is an independent predictor of cognitive dysfunction and dementia (24–28). These studies, many of which were performed in epidemiologic cohorts including the Framingham Heart Study, the Maastricht study, and the PARTAGE study, found strong associations between stiffness and cognitive impairment. Interestingly, in the Maastricht study, the authors found a strong association with central stiffness (−0.018 SD [95% CI, −0.036 to −0.000]), but not with carotid stiffness (24). In addition, there are multiple studies establishing a cross-sectional association of central arterial stiffness to cognitive decline (29–32). Though there is relatively strong data supporting a link between central arterial stiffening to cognitive dysfunction, there is less supporting evidence between carotid stiffness and dementia with some studies supporting this association (Table 2) (33–38), and other studies showing no association, after adjusting for other confounders (24, 67, 68). This is certainly an area worthy of future investigation to further our understanding of the role of carotid stiffness, an asymptomatic marker of vascular aging, to the development of dementia. Future longitudinal prospective cohort studies may be able to aid in clarifying this potential association.

**Table 2:** Detailed study description for each feature of atherosclerosis.

**Atherosclerosis feature**	**Key studies evaluating link to cognitive dysfunction**
Carotid stiffness	Among 1,662 women (median baseline age = 41), greater carotid stiffness was associated with greater decline in neuropsychological test scores over 10-year follow-up (33)
	Higher carotid stiffness and lower compliance were associated with slower processing speed in 32 middle aged adults (mean age 64.2) (34)
	Higher carotid stiffness is associated with lower MMSE scores in 308 adults (mean age 63) without known vascular disease (35)
	Lower carotid artery stiffness in endurance athletes is associated with better neuropsychological outcomes (36)
	Higher carotid stiffness index is associated with reduced executive functioning processing speed in smokers (37)
	Carotid stiffness is associated with worse cognitive performance, primarily in processing speed and executive function and attention (38)
Carotid Intima-media thickening	In 8,208 participants (mean age 49.6 years), CIMT was inversely associated with memory function (39)
	CIMT ≥0.9 mm is independently associated with lower cognitive performance in 245 patients with asymptomatic HIV (40)
	In 3,227 participants (mean age 57.9 years), larger CIMT was associated with lower MMSE scores after adjustment for confounders (41)
	In 231 older adults, CIMT was associated with mild cognitive impairment after multivariate adjustment (42)
	In 1,826 patients with acute ischemic stroke, those with highest CIMT quartile were more likely to have cognitive impairments compared to the lowest IMT quartile (43)
	In 348 non-demented participants (mean age 71.7 years), greater baseline CIMT was independently associated with mild cognitive impairment and dementia diagnosis during a 5-year follow-up period (44)
	In 251 participants (mean age 78 years), there was a significant association between CIMT and change in executive functioning over a mean 2.3 year follow-up (45)
	Higher CIMT was associated with worse episodic memory after adjustment for vascular risk factors in 1,166 stroke-free participants being followed for about 5 years (46)
Flow-limiting stenosis	Asymptomatic participants with carotid stenosis had significantly lower levels of performance in tests of attention, psychomotor speed, memory, and motor functioning compared to those without stenosis (47)
	High-grade left carotid artery stenosis is associated with cognitive impairment and cognitive decline (48)
	Asymptomatic patients (*n* = 548) with high-grade carotid stenosis had worse performance on neuropsychological testing than healthy controls (49)
	Asymptomatic patients with high-grade carotid stenosis were found to have high rates of cognitive deterioration (51)
	Those with asymptomatic bilateral high-grade carotid stenosis had high risk of developing cognitive impairment (52)
	Those with severe asymptomatic unilateral carotid stenosis were found to have poor performance on neuropsychological exams (53)
High-risk plaque features	In a cross-sectional study of 284 patients with dementia, higher plaque volume is strongly associated with dementia (60)
	In 1,279 participants, those with carotid plaque (<40% stenosis) had a moderate association with poor cognitive performance (61)
	In 406 patients followed for 1 year, those with higher carotid plaque index were more likely to develop dementia (62)
	In 210 participants, the presence of carotid plaque was associated with abnormal cognitive function adjusting for confounders (64)
	In 2015 participants, those with carotid plaque were more likely have poor cognitive function (66)

## Carotid Intima-Media Thickening and Cognitive Impairment

### Definition

Another marker of asymptomatic carotid atherosclerosis is thickening of the carotid intima-media. This subtle thickening of the arterial wall measured in either the distal common carotid artery or proximal internal carotid artery using ultrasound is a marker of subclinical atherosclerosis. Similar to arterial stiffness, both hypertension and smoking, along with other vascular risk factors, are known associations to CIMT. Since carotid intima-media thickening (CIMT) is a precursor for the development of atherosclerotic plaque, it is often considered an imaging marker of generalized cardiovascular risk. Though this thickening is asymptomatic, it is associated with cognitive dysfunction and dementia, along with stroke and overall increased mortality risk (47, 60, 69, 70).

### CIMT and Imaging Markers Associated With Cognitive Impairment

**Figure 2:**
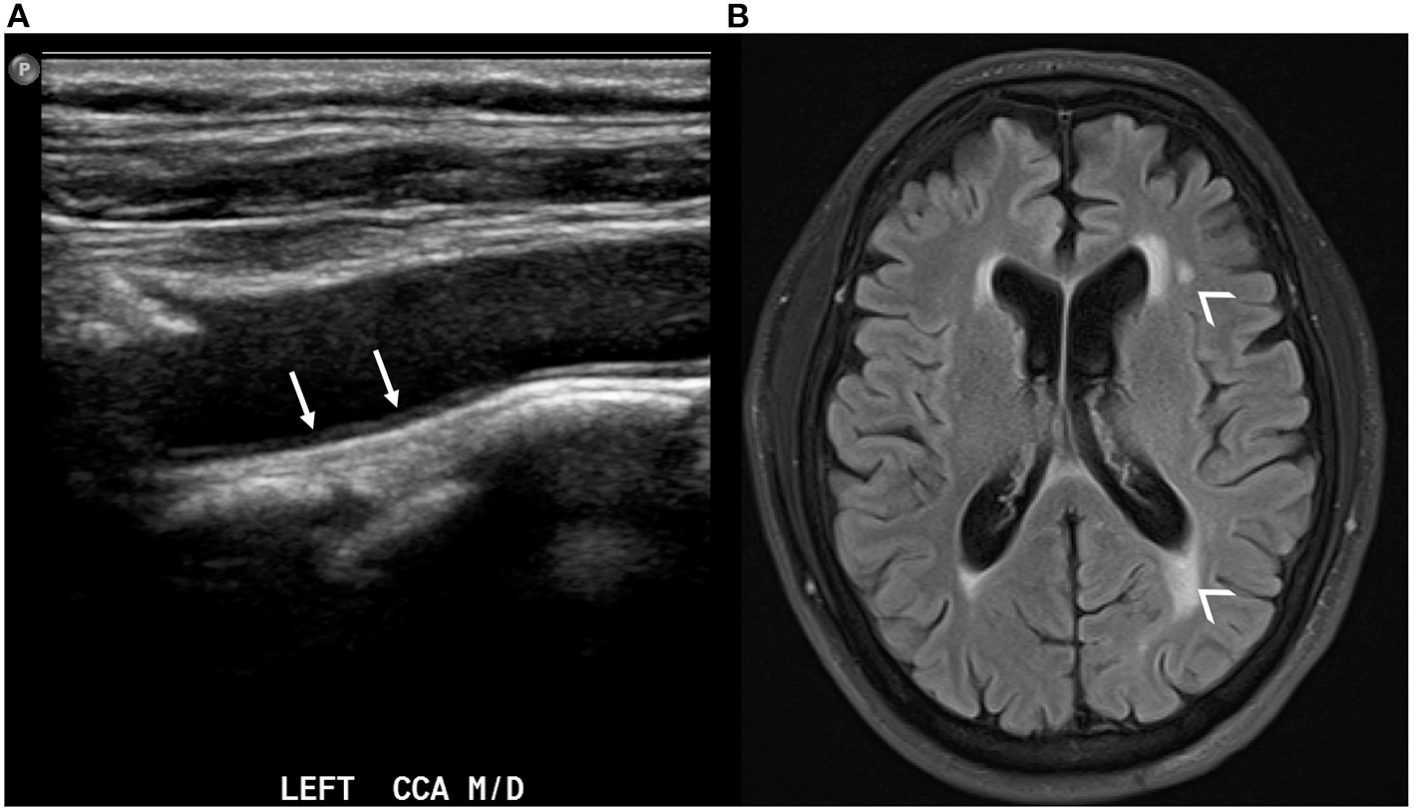
Carotid ultrasound demonstrating thickening of the intima-media in the left common carotid artery [**(A)**, white arrows]. The same patient also has evidence of white matter hyperintensities in the periventricular white matter on axial T2 FLAIR brain MRI [**(B)**, small arrowheads].

There is evidence of an association of baseline increased CIMT to brain imaging markers of cerebral small vessel disease including white matter hyperintensities, covert brain infarctions, cerebral microbleeds, and also lower total brain volumes (71–76) (Figure 2). Specifically, a systematic review and meta-analysis of nine studies found that CIMT was associated with white matter hyperintensities [odds ratio (OR) 1.42, 95% CI 1.22–1.66, *p* < 0.0001], covert brain infarctions (OR 1.89, CI 1.46–2.45, *p* < 0.0001) (74). As for the association of CIMT with brain volume, there is evidence of lower total brain volume (−0.05 per SD, *P* < 0.05) in the Framingham Heart Study (77) and lower total brain and cortical gray matter (−0.29 per SD) in the SMART-MR study cohort (72). In addition, there is evidence that the progression of CIMT over time is also associated with lower hippocampal volumes in the Framingham Heart Study (78). As discussed previously, these imaging markers of cerebral small vessel disease and brain aging are independently associated with cognitive dysfunction and dementia.

### CIMT and Cognitive Impairment

Several studies have evaluated the association between CIMT on ultrasound and the future development of dementia and cross-sectional association with cognitive function. Several studies from epidemiologic cohorts have found a positive association between increased CIMT at baseline and future development of cognitive decline (44–46). These studies followed cohorts of participants over time and found that having higher baseline CIMT on ultrasound was correlated with poorer future performance on cognitive testing and increased rates of dementia diagnoses. Hazard ratio for development of cognitive impairment based on elevated CIMT was 1.251 (95% CI 1.006–1.555, *p* = 0.044) in the Korean Longitudinal Study on Health and Aging after adjusting for basic demographics and baseline cognition (44). Further, in the Northern Manhattan Study, a cohort study of stroke-free participants, found that those with greater CIMT had worse performance on cognitive testing (ß = −0.60, *p* = 0.04 for episodic memory) (46). There are also many studies demonstrating a cross-sectional association between increased CIMT and poorer cognitive function (39–43).

### Potential Mechanism

The exact pathophysiologic mechanism underpinning this association is unclear at this time, though it may be similar to the proposed mechanisms for arterial stiffness. Since CIMT is thought to be marker for systemic atherosclerosis, it may not necessarily be a direct contributor to cognitive dysfunction, but rather reflect general cardiovascular risk. Further evaluation of this association is necessary in order to identify potentially modifiable vascular contributions to cognitive dysfunction.

## Carotid Stenosis and Cognitive Impairment

### Definition

Flow-limiting carotid stenosis is a well-documented risk factor for stroke and is an established indication for carotid revascularization. Severe carotid stenosis is traditionally defined as 70–99% narrowing of the vessel lumen by various measuring methods, most commonly the North American Symptomatic Carotid Endarterectomy Trial (NASCET) method in the United States (79). Moderate stenosis is defined as 50–70% stenosis. The degree of stenosis is a leading risk factor in the development of ischemic stroke with annual rates of ischemic stroke estimated to be around 1% (80). Though not nearly as well-studied, some have found that the annual rate of dementia in the presence of severe carotid stenosis to be around 1 (55).

### Potential Mechanisms

The main mechanism by which carotid stenosis is hypothesized to contribute to cognitive impairment is via hypoperfusion (81). By limiting the flow to the brain parenchyma, some hypothesize that this may lead to end-organ damage, parenchymal atrophy, and neurodegneration. Cerebral hypoperfusion is thought to accelerate amyloid and tau deposition, which is a potential link between flow-limitation and cognitive dysfunction (82, 83). Other potential mechanistic explanations include via covert brain infarctions from embolization, which may act as an intermediate step between stenosis and cognitive dysfunction. Similar to many other features of carotid atherosclerosis, it is difficult to disentangle the complex associations between carotid stenosis, other vascular risk factors, and other findings of cerebral parenchymal damage. One potential method for clarifying these associations is by evaluating individuals with unilateral stenosis to determine if there are varying degrees of parenchymal damage downstream from the affected side. Currently, the exact mechanism by which flow-limiting stenosis contributes to cognitive dysfunction is not clearly established.

### Stenosis and Cognition

Several studies have shown an association with carotid stenosis and poorer performance on cognitive testing (47–52), while others have failed to find an association. There are several studies finding that asymptomatic individuals with severe carotid stenosis with evidence of altered perfusion are more likely to develop cognitive impairment (51, 84, 85). In one of the largest studies evaluating this association in over 4,000 participants, the authors found that high-grade carotid artery stenosis was associated with cognitive impairment (OR 6.7, 95% CI 2.4 to 18.1) and cognitive decline (OR 2.6, CI, 1.1 to 6.3) (48). Some have found that left sided carotid stenosis is more likely to result in cognitive dysfunction than right-sided carotid stenosis, indicating that carotid stenosis may be an independent risk factor for cognitive impairment (48, 53). A systematic review and meta-analysis including over 760 subjects with asymptomatic carotid stenosis found an association between the presence of carotid stenosis and cognitive impairment (54).

Several studies have attempted to isolate the effect of carotid stenosis by assessing for changes in cognitive function after carotid endarterectomy with conflicting results (56). Overall, the majority of studies have found an improvement in cognition after CEA, but there are many studies showing either no change, and even a deterioration in cognitive function. In a large study of patients with asymptomatic carotid stenosis, there was no difference in mini-mental status examination scores in those who received medical therapy compared to those who underwent CEA (57). Another study based on patients from a randomized controlled trial with severe carotid stenosis without history of stroke or known dementia (ACST-1) found that carotid endarterectomy had no significant effect on the incidence of dementia (55). There is less data regarding cognitive changes after CAS, however, there are similarly mixed results with some studies showing an improvement in cognition after undergoing CAS (56, 58, 59).

### Stenosis and Imaging Markers Associated With Cognitive Impairment

There is strong evidence that the presence of flow-limiting stenosis results in both cerebral atrophy as well as other markers of subclinical vascular injury, such as white matter hypterintensities and CBIs. There is evidence that severe carotid stenosis is associated with progression of brain atrophy, while the same link was not as pronounced in those with moderate stenosis (72). Further, there is strong evidence that carotid stenosis is associated with markers of parenchymal damage, including white matter hyperintensities and microstructural damage to both gray and white matter (86–88). In addition, there is strong evidence of an association between carotid stenosis and downstream CBI with a systematic review and meta-analysis of 11 studies reporting an OR of 2.78 (95% CI, 2.19 to 3.52, *p* < 0.0001) (89). Further, studies demonstrate that there are asymmetries in prevalence of CBI in cerebral hemispheres downstream from severe carotid stenosis, specifically with more cortical CBIs downstream from stenosis (90).

This role of flow-limiting stenosis in the development of dementia is an area worthy of further investigation. Though CEA and CAS procedures are primarily performed for stroke risk reduction, the potential added bonus of improved cognition may alter the risk calculus when identifying patients for carotid revascularization.

## Carotid Plaque and Cognitive Impairment

### Definition

Carotid plaque is a specific marker of advanced atherosclerosis usually found in the carotid bifurcation. There are many different features of the plaque itself that carry varying degrees of associated embolic risk. There has been increased attention on individual plaque components and plaque volume in addition to the degree of stenosis when assessing stroke risk from carotid atherosclerotic disease. There is strong evidence that certain plaque components are more strongly associated with future and recurrent stroke, including intraplaque hemorrhage, lipid-rich necrotic core, and plaque ulceration (91). Many of these specific plaque elements are often more strongly associated with stroke than degree of stenosis which has led to a paradigm shift in stroke risk assessment (92). Though the major concern for carotid plaques is their leading to symptomatic strokes and/or transient ischemic attacks, many of these high-risk plaque features are also seen in asymptomatic individuals.

Though there is a strong association with high-risk plaque elements to stroke, there are fewer studies evaluating the association of plaque with the development of cognitive dysfunction and dementia. The current literature has shown mixed results with respect to the association of vulnerable plaque components and cognitive dysfunction.

### Plaque and Cognition

Some studies have evaluated the association of increased plaque volume to performance on cognitive examinations and have found that there is worse performance on cognitive testing with higher plaque volume, even when accounting for education level and other confounding (60–66). For example, a study evaluating high risk plaque features on ultrasound found that those with more plaques were more likely to have poor performance on cognitive testing, including mini-mental status examinations (OR 1.72, 1.00–2.96) (61). Most of the studies evaluating this association used either ultrasound or CT imaging techniques to evaluate plaque. Other studies have shown no significant difference in cognitive function when accounting for other cardiovascular risk factors (93).

### Plaque and Imaging Markers Associated With Cognitive Impairment

**Figure 3:**
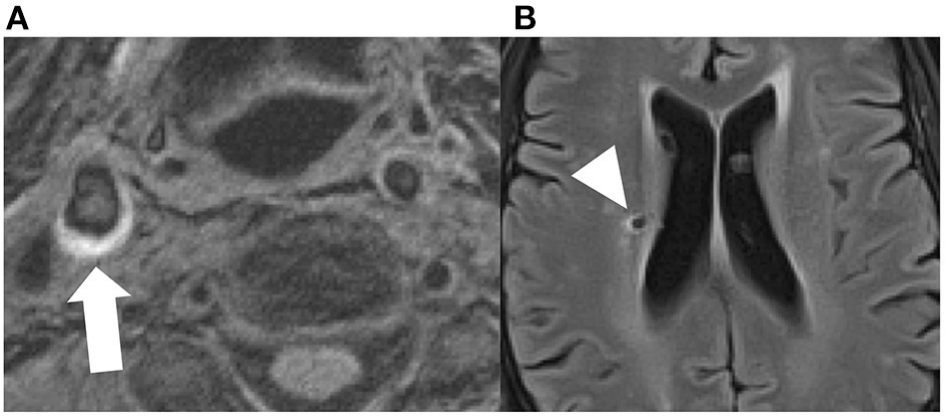
Axial MPRAGE sequence demonstrating T1 hyperintensity in the right carotid bifurcation, compatible with intraplque hemorrhage [**(A)**, white arrow]. The same patient had a small covert brain infarction in the right periventricular white matter [**(B)**, arrowhead].

There is evidence that vulnerable plaque features are associated with other markers of neurodegeneration and subclinical vascular injury. For instance, there is evidence that vulnerable plaque features contribute to cortical micro-infarcts detected on MR which are in turn associated with poor cognitive function (94). There are other studies which demonstrate an association with white matter hyperintensities and CBIs as well (74), though there are few studies explicitly looking at specific plaque features (Figure 3).

### Potential Mechanisms and Future Directions

The exact mechanism behind this potential association is unclear at this time. Whether high risk plaque is directly associated with dementia due to repeated microembolic phenomena is not well established. Though there is evidence that high-risk plaque lead to increased risk of stroke, it is unclear if these plaque features may also contribute to microembolic phenomenon contributing to cognitive dysfunction. Other studies have found that there are increased markers of subclinical vascular injury which are associated with high-risk plaque features, including covert brain infarctions, cerebral microbleeds, and white matter hyperintensities which are independently associated with cognitive dysfunction. At this time, this exact association is unclear and further studies would be helpful in elucidating the exact contribution of high-risk plaque features to cognitive dysfunction and dementia.

## Conclusion

Ranging from increased stiffness of the arterial wall to complex atherosclerotic plaques prone to rupture, there is a wide variety of manifestations of carotid disease. All of these manifestations can be seen in asymptomatic individuals. Though there has been a strong association between many features of carotid atherosclerosis and stroke, there has been less attention on the link to cognitive dysfunction. In this review, we present the existing evidence supporting this potential link.

There are many risk factors associated with the development of the presented types of carotid atherosclerosis, ranging from carotid stiffness to complex, vulnerable plaque components, including smoking and hypertension. A potential method for mitigating the association of asymptomatic carotid atherosclerosis and the development of cognitive impairment is to target these known contributors to atherosclerosis.

In the studies cited in this review, there are many definitions and diagnostic criteria for various types of cognitive dysfunction and dementia including mild cognitive impairment, vascular dementia, and Alzheimer's and Alzheimer's-related dementia. Future studies are needed with more streamlined diagnostic criteria and biomarker validation for the various forms of cognitive dysfunction. Future prospective longitudinal studies are necessary to further elucidate this relationship. As more studies confirm this link, we may expect changes to the risk-benefit assessment of pursuing surgical intervention in asymptomatic individuals with certain types of carotid artery atherosclerosis. In addition, with stronger scientific support, more targeted preventative strategies, including stringent medical management, could be considered directed at the development of carotid atherosclerosis as a means of dementia prevention. Currently, there is formal guidance not to perform routine screening for extracranial carotid plaque in individuals who are asymptomatic from the US Preventive Task Force. Though there is evidence of a potential link between asymptomatic carotid disease and cognitive impairment, the existing data is not strong enough at this time to reverse this recommendation. Further prospective observational studies confirming the link between carotid disease and cognitive impairment are necessary before making this recommendation. Additional evidence could warrant altering this recommendation including a randomized controlled trial comparing stringent medical management to those with less standard of care treatment with asymptomatic carotid artery disease showing that changes to medical management may decrease risk of cognitive impairment.

## Author Contributions

AG made substantial contributions to the conception or design of the work, drafted the work and revised it critically for important intellectual content, provided approval for publication of the content, and agreed to be accountable for all aspects of the work in ensuring that questions related to the accuracy or integrity of any part of the work are appropriately investigated and resolved. All authors contributed to the article and approved the submitted version.

## Funding

HB is in part supported by the University of Utah Program in Personalized Health and National Center for Advancing Translational Sciences of the National Institutes of Health under Award Number 1UL1TR002538.

## Author Disclaimer

The content is solely the responsibility of the authors and does not necessarily represent the official views of the National Institutes of Health.

## Conflict of Interest

The authors declare that the research was conducted in the absence of any commercial or financial relationships that could be construed as a potential conflict of interest.

## Publisher's Note

All claims expressed in this article are solely those of the authors and do not necessarily represent those of their affiliated organizations, or those of the publisher, the editors and the reviewers. Any product that may be evaluated in this article, or claim that may be made by its manufacturer, is not guaranteed or endorsed by the publisher.
